# Establishment of prostate cancer spheres from a prostate cancer cell line after phenethyl isothiocyanate treatment and discovery of androgen-dependent reversible differentiation between sphere and neuroendocrine cells

**DOI:** 10.18632/oncotarget.8440

**Published:** 2016-03-28

**Authors:** Yamei Chen, Shundong Cang, Liying Han, Christina Liu, Patrick Yang, Zeeshan Solangi, Quanyi Lu, Delong Liu, J.W. Chiao

**Affiliations:** ^1^ Department of Medicine, New York Medical College, Valhalla, NY 10595, USA; ^2^ Department of Oncology, Henan Province People's Hospital, Zhengzhou University, Zhengzhou, China; ^3^ Department of Pathology, New York Medical College, Valhalla, NY 10595, USA; ^4^ Department of Hematology, Zhongshan Hospital, Xiamen University, Xiamen, Fujian, China; ^5^ Department of Oncology, The First Affiliated Hospital of Zhengzhou University, Zhengzhou, China

**Keywords:** prostate cancer, stem cells, sphere, epigenome, androgen

## Abstract

Prostate cancer can transform from androgen-responsive to an androgen-independent phenotype. The mechanism responsible for the transformation remains unclear. We studied the effects of an epigenetic modulator, phenethyl isothiocyanate (PEITC), on the androgen-responsive LNCaP cells. After treatment with PEITC, floating spheres were formed with characteristics of prostate cancer stem cells (PCSC). These spheres were capable of self-renewal in media with and without androgen. They have been maintained in both types of media as long term cultures. Upon androgen deprivation, the adherent spheres differentiated to neuroendocrine cells (NEC) with decreased proliferation, expression of androgen receptor, and PSA. NEC reverse differentiated to spheres when androgen was replenished. The sphere cells expressed surface marker CD44 and had enhanced histone H3K4 acetylation, DNMT1 down-regulation and GSTP1 activation. We hypothesize that PEITC-mediated alteration in epigenomics of LNCaP cells may give rise to sphere cells, whereas reversible androgenomic alterations govern the shuttling between sphere PCSC and progeny NEC. Our findings identify unrecognized properties of prostate cancer sphere cells with multi-potential plasticity. This system will facilitate development of novel therapeutic agents and allow further exploration into epigenomics and androgenomics governing the transformation to hormone refractory prostate cancer.

## INTRODUCTION

Prostate cancer initially responds to androgen deprivation therapy but eventually becomes refractory to hormone therapy. These hormone refractory cancer cells grow independently of androgen, become highly aggressive, and metastasize quickly. Hormone refractory prostate cancer leads to high mortality since there lacks effective therapy. One major reason is that the precise molecular mechanisms responsible for the cancer progression remain unclear. The most prominent mechanisms postulated to date involve the androgen receptor (AR). They include AR gene mutation, excessive recruitment of AR transcription co-regulators [1, 2], non-AR-related bypass pathways [3], and epigenetic alterations including hypermethylation of AR promoter [4]. Other mechanisms postulated include the involvement of prostate cancer stem cells (PCSC). Epithelial androgen-independent prostate cancer stem cells or AR negative stem cells have been hypothesized to be present in the prostate cancers which become enriched by androgen deprivation therapy [5, 6].

Prostate cancer stem cells have been a subject of our research. We reported in 1999 that the androgen-dependent human prostate cancer cells LNCaP could be induced to form neuroendocrine cells by interleukin 1. These neuroendocrine cells were significantly attenuated in proliferation and in PSA production [7]. Similarly, Bang *et al* reported that LNCaP cells underwent neuroendocrine cell differentiation mediated by cyclic AMP analogues [8]. Clinical cases of transformation of prostate adenocarcinoma to neuroendocrine cancer type have been reported [9]. Prostate cancer stem cells (PCSC) have been shown in spheres from prostate cancer cell line DU-145 [10], from castration-resistant LNCaP cell line [11], and also from xenografts of prostate cancer specimen [12]. Spheres were obtained in cell cultures primarily with the aid of special culture media or Matrigel which are rich in growth factors. The characteristics of PCSC within the spheres, the mechanisms by which they renew and differentiate, as well as their androgen dependency have yet to be defined.

Epigenetic agents have been shown to be potentially effective treatments for prostate cancer [13, 14]. We have demonstrated that phenethyl isothiocyanate (PEITC) is an epigenetic agent with dual activity to inhibit DNA hypermethylation and histone deacetylation [15, 16]. In this study, LNCaP cell spheres were established after PEITC treatment. The sphere cells expressed cancer stem cell marker CD44 and had epigenomic alterations distinct from the parental LNCaP cells. The sphere cells can self-renew with androgen and also in androgen-deprived condition. Furthermore, the sphere cells were capable of differentiating to neuroendocrine cells (NEC) when androgen was deprived. The NEC could reverse-differentiate to spheres when androgen was replenished.

## RESULTS

### Establishment and structure of the prostate cancer spheres

**Figure 1:**
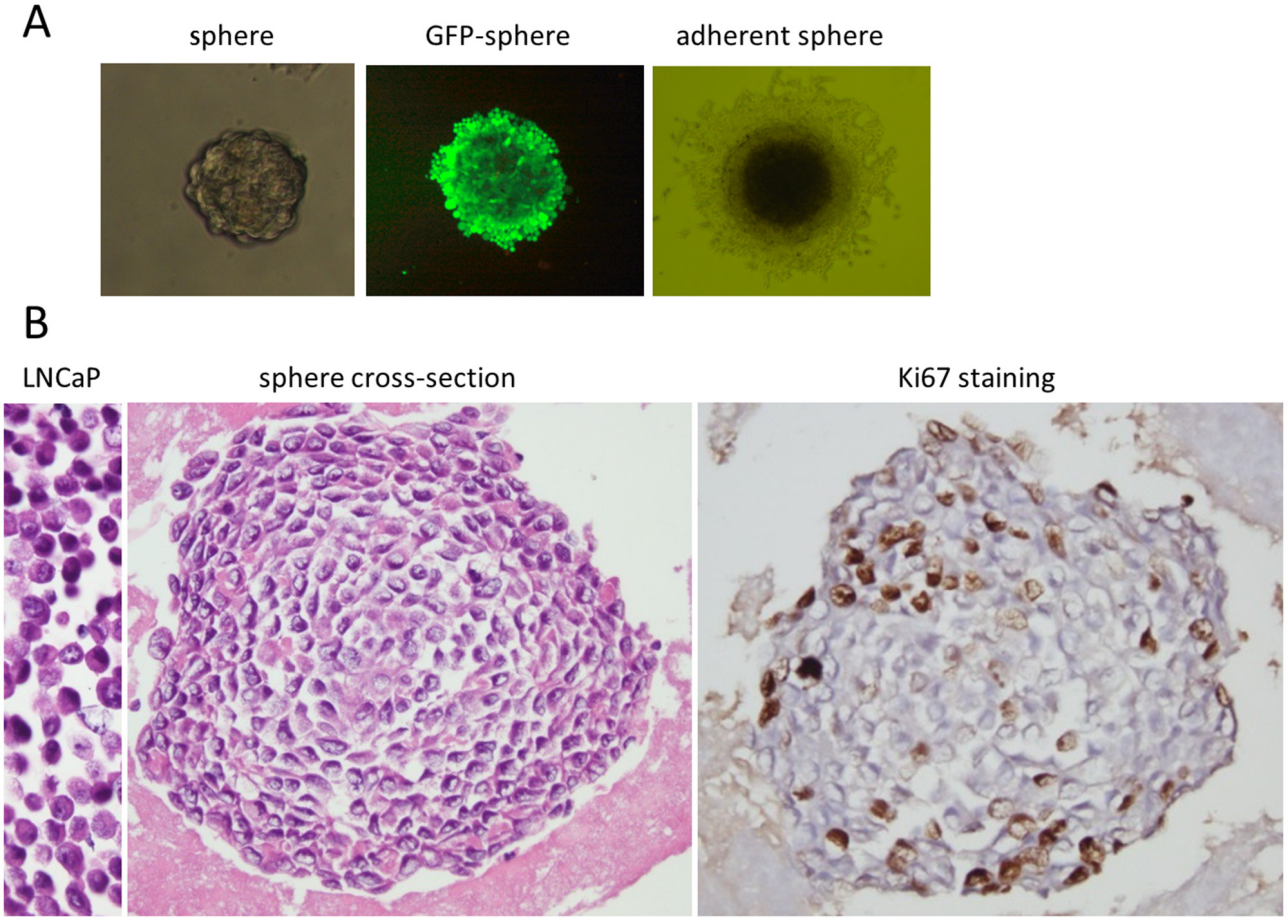
Structure of LNCaP prostate cancer spheres **A.** Representative bright-field images of LNCaP cell spheres cultured in RPMI-1640 medium with 10% FBS. From left to right are floating 3D-sphere, 400x magnifications; fluorescent image of a GFP-labeled LNCaP cell sphere, 400x magnifications; and an adherent sphere, 200x magnifications. **B.** The left is an image of H & E stained paraffin embedded LNCaP cells; the center is a cross section of a sphere, paraffin embedded and H& E stained; and the right is cross section of a sphere, paraffin embedded and immuno-stained for Ki67 expression. The dark spots are the Ki67 positive cells. Images are 400x magnifications.

LNCaP cells were exposed to PEITC at various concentrations and the development of spheres was investigated. PEITC at 4 μM was found to be optimal for mediating the formation of floating spheres after 4-7 days. There were no floating spheres in the control cultures of LNCaP cells without PEITC. The spheres were enriched with continued exposure to 3 μM PEITC for 8-12 days. With three different lots of LNCaP cell cultures examined, the number of spheres obtained were approximately 0.46% (ranged from 0.18%-0.62%) of the starting LNCaP cells. Each floating sphere was initially composed of several cells, which grew larger in size to form the characteristic 3D-clusters (Figure 1A left, sphere). To demonstrate that the PEITC-induced spheres were indeed derived from the LNCaP cells, a separate LNCaP cell line expressing green fluorescent protein (GFP-LNCaP) was examined for sphere development. As shown in Figure 1A (center, GFP-sphere), the floating spheres with GFP fluorescence were reproducibly developed from the GFP-LNCaP cell line following the same PEITC treatment protocol. This indicated that the spheres were formed *de novo* from the LNCaP cells, not due to contamination by other cells. Floating spheres could be maintained as long term culture in flasks with ultralow attachment, in RPMI-1640 medium and 10% regular FBS without PEITC, and they do not revert back to parental LNCaP cells. The spheres could form adherent culture in regular culture flasks, and had cells migrated outward from the spheres (Figure 1A right). The spheres were maintained in the long-term culture with morphology distinct from the LNCaP cell monolayer culture.

Furthermore, we have also examined the sphere formation properties of two other prostate cancer cell lines, PC-3 and DU-145. The sphere formation efficiencies and sizes of spheres were recorded (Supplementary Table S1). The quantity of spheres was approximately less than 0.05% of the starting PC-3 cells, approximately 1/10^th^ of the sphere formation efficiencies from LNCaP and GFP-LNCaP cell lines. Under similar conditions, no sphere formation was observed from DU-145. Thus, the sphere formation property appears to be intrinsic to the individual cell lines.

To examine the 3D-structure of the spheres, the spheres were prepared in paraffin blocks for H & E staining. Figure 1B shows a typical cross section of the LNCaP spheres (Figure 1B, sphere cross-section). Cells were shown to line up concentrically, with cell layers expanding circularly outward from the center. The outer cell layers were more tightly compacted than those in the interior. The sphere cells were relatively smaller than the parental LNCaP cells (Figure 1B, LNCaP), and displayed different morphology with oval shaped nuclei, high nuclear-cytoplasmic ratio, irregular nuclear contour and prominent nucleoli (Figure 1B). The LNCaP cells prepared identically had eccentric nuclei, irregular nuclear outlines and intermediate cytoplasm (Figure 1B left, LNCaP). The proliferation of the sphere cells was evaluated by immunostaining of Ki67, a marker of cell proliferation. Ki67 expression was found mainly in the periphery of the spheres, as shown in a typical staining (Figure 1B, Ki67 staining). The peripheral location of Ki67 expression and the concentric cell layers suggested a spherical growth pattern. To assess the self—renewal potential of the spheres, single cells from spheres were seeded with limiting dilution in medium with regular FBS. New spheres were regularly formed, which could be maintained for multiple generations and have been kept as long term cultures for more than two years. In these cultures the adherent spheres may detach and re-attach to the culture vessels during culture passage.

### Spheres grew slower in androgen-deficient medium

**Figure 2:**
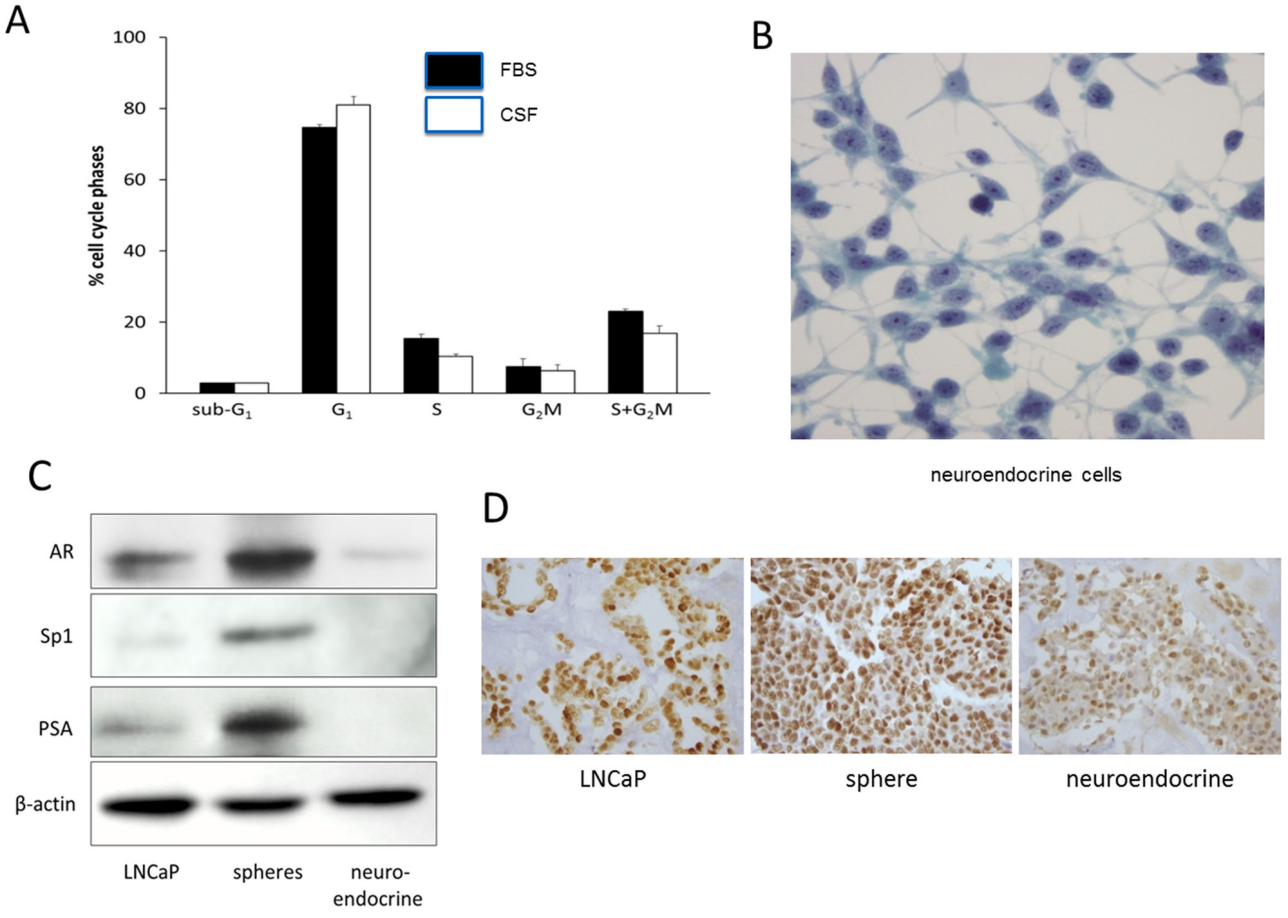
Growth and differentiation of LNCaP-derived sphere cells **A.** Cell cycle analysis of sphere cells. LNCaP sphere cultures maintained in RPMI-1640 medium with 10% regular FBS (solid column) or with10% charcoal-stripped FBS (CSF) (open column) were analyzed for cell cycle phase distribution by flow cytometry. Vertical bars are mean ± SD of 3 separate experiments. **B.** Neuroendocrine cells developed from adherent sphere cells in medium with 10% CSF on chamber slides, Pap stained, 400 × magnifications. **C.** Western blots showing the expression of androgen receptor (AR), Sp1, and PSA in LNCaP cells (maintained in regular FBS medium), spheres (maintained in regular FBS medium), and neuroendocrine cells (maintained in CSF medium). β-actin was used as a loading control. **D.** Immunohistochemical staining of AR in paraffin embedded preparations. From left to right are LNCaP cells, spheres (regular FBS medium), and neuroendocrine cells (CSF medium), 400x magnifications.

To determine the effect of androgen on the sphere cell growth, the spheres were cultured in medium with 10% steroid hormone-depleted charcoal dextran-stripped FBS (CSF) which contains lower than castrate level of testosterone [17]. The sphere cultures in the CSF medium maintained good viability but grew slower than the spheres kept in the regular FBS medium. The spheres have been maintained in both types of media as long term cultures, and their proliferation was evaluated with cell cycle phase progression using flow cytometry. Figure 2A shows that the sphere culture in CSF medium (open bar) had significantly fewer replicating cells in S and G_2_M phases (19.2 ± 2.3%) than those spheres grown in the regular FBS medium (solid bar) (26.1± 1%)(*P*=0.022).The results indicated that the spheres could self-renew in the presence of androgen, but at a significantly slower rate in the absence of androgen.

### Differentiation of sphere cells to neuroendocrine cells

We next examined the differentiation of sphere cells in the presence and absence of androgen. The monolayer cells spreading from adherent spheres underwent gradual morphological changes in the CSF medium, as compared to sphere cells cultured with regular FBS medium. The cells became elongated and displayed dendritic projections which were seen to bridge with other cells (Figure 2B). The cells had round nuclei, a high nuclear-cytoplasmic ratio, and prominent nucleoli (Figure 2B). This morphology resembles the characteristics of neuroendocrine cells (NEC) [7, 8]. They were further examined for the presence of neuroendocrine cell markers by immunohistochemical staining. As summarized in Table 1, these cells were positive for serotonin, neuro-specific enolase, and pan-cytokeratin, the biochemical hallmarks of NECs. These markers were at background levels in the LNCaP cells and the spheres (Table 1).

**Table 1:** Expression of neuroendocrine cell markers

Markers	Staining intensity		
	LNCaP	Spheres	Neuroendocrine cells
Pancytokeratin	+/−	-	+++
Neuro-specific enolase	+/−	−	+++
Serotonin	−	−	+

Definition of staining intensity: +/− : focally and weakly positive; − : negative; + : weakly positive; +++: strongly positive.

Since NEC differentiation was induced in medium with charcoal-stripped FBS (androgen-depleted), the involvement of androgen receptor (AR) in the differentiation was investigated. AR expression in the NEC was at the background level, approximately 2.8 folds lower than that of the LNCaP cells, and approximately 6.1 folds lower than that of sphere cells (Figure 2C). We also examined the expression of the transcription factor Sp1, which is known to mediate AR transcription [18, 19]. Sp1 mirrored the expression pattern of AR, with the LNCaP reduced approximately 5.7 folds than the spheres while the NEC expressed it only at the background level (Figure 2C). The level of PSA, which is a down-stream target gene of AR, also followed the expression pattern of AR, in that PSA in the LNCaP was approximately 7.5 folds lower than that of the sphere cells, and PSA in NEC was not detectable (Figure 2C). The reduced AR expression in the NEC was further verified by immunohistochemical staining of the cells prepared in paraffin block. Figure 2D shows that the NEC had a much reduced staining intensity of AR as compared to the LNCaP cells and the sphere cells. These results suggest that reduction of AR expression may be an important mechanism for the differentiation of spheres to NEC.

### Reversible differentiation between spheres and neuroendocrine cells

**Figure 3:**
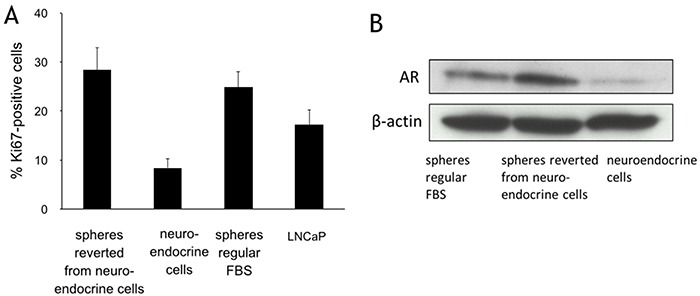
Reversible differentiation between LNCaP spheres and neuroendocrine cells **A.** Immunohistochemical staining of Ki67 expression in LNCaP cells (maintained in regular FBS medium), spheres (regular FBS medium), neuroendocrine cells (CSF medium), and spheres reverse differentiated from the neuroendocrine cells (maintained in regular FBS medium). Columns and vertical bars represent means ± SD of 4 independent experiments. **B.** Western blots for androgen receptor (AR) expression in the neuroendocrine cells (CSF medium), spheres reverse differentiated from the neuroendocrine cells (maintained in regular FBS medium), and spheres (maintained in regular FBS medium). β-actin was used as a loading control.

When CSF culture medium was replaced with regular FBS medium for 7-14 days, the neuroendocrine cells gradually lost the dendritic morphology, and spheres reappeared on the monolayer, implying that androgen may propel neuroendocrine cells back to spheres. To further characterize the effect of androgen on the reformation of spheres from NEC, dihydrotestosterone (DHT) was added to the CSF culture medium. Addition of DHT from 1-80 nM induced sphere formation in a concentration-dependent manner. DHT at 10 nM was optimal, with spheres forming in 3-5 days. These spheres had significantly more replicating cells than the NEC, as indicated by Ki67 staining (Figure 3A). The spheres had approximately 28.3 ± 4.2% Ki67 positive cells, a major increase from 8.4 ± 2.1% of the NEC. The Ki67 level in the DHT-driven spheres was similar to that of the spheres grown in regular FBS medium (25.8 ± 3.6%), but more than those in the parental LNCaP cells (16.2 ±3.1%). AR expression in the reverted spheres was significantly increased, to approximately 5.5 folds more than that in the NEC (Figure 3B). The morphological and biochemical changes corroborated the androgen-dependent reversibility between the spheres and NEC. The plasticity of the sphere cells supports the notion that these spheres contain prostate cancer stem cells.

### Distinct phenotype and invasive activity of spheres and neuroendocrine cells

**Figure 4:**
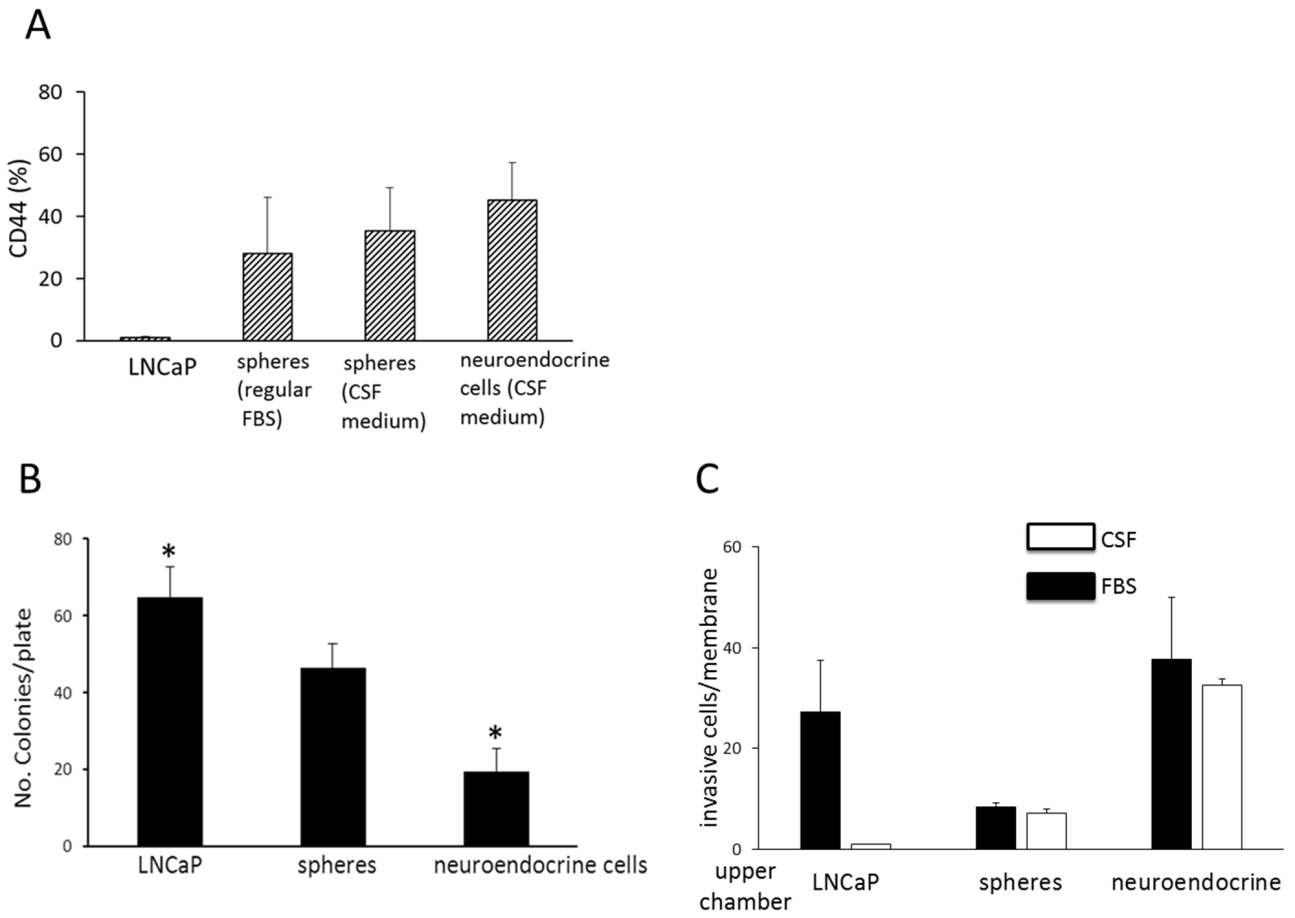
Phenotype and invasive activity of sphere and neuroendocrine cells **A.** CD44 expression. This figure shows the proportion of CD44 positive cells among LNCaP cells maintained in regular FBS medium, sphere cells maintained in regular FBS medium, sphere cells maintained in CSF medium, and neuroendocrine cells maintained in CSF medium, as determined by a flow cytometric method. Columns and vertical bars represent the means ± SD of four independent experiments. **B.** Soft-agar gel colony formation assay showing number of colonies per plate of LNCaP cells (prepared in regular FBS medium), spheres (prepared in regular FBS medium), and neuroendocrine cells (in CSF medium). Columns and vertical bars are means ± SD of 4 separate experiments. * indicates statistically significant difference in colony numbers between LNCaP and neuroendocrine cells, *P*<0.05. **C.** Matrigel migration assay. This figure shows the number of invasive cells per membrane of LNCaP cells (maintained in regular FBS medium), spheres (regular FBS medium), and neuroendocrine cells (CSF medium) from the Matrigel chamber invasion assay. Chemoattractant used in the lower chamber: solid bar (■) indicates regular FBS; open bar (o) indicates charcoal-stripped FBS (CSF). Columns and vertical bars indicate the means ± SD of four separate experiments.

Prostate cancer stem cells have been described to express surface marker CD44 [20, 21]. Flow cytometric analyses demonstrated that LNCaP cells had background level of CD44 staining, whereas approximately 28.1 ± 16 % of the sphere cells grown in regular FBS medium were positive for CD44 (Figure 4A). The sphere cells maintained in CSF medium were approximately 35.2 ± 12% positive for CD44, and the NEC approximately 42.2 ± 12% CD44–positive (Figure 4A). CD44 may thus be a sphere marker distinct from the parental LNCaP cells.

Anchorage-independent growth is one of the hallmarks of cell transformation and tumorigenic potential. To characterize whether the spheres and NEC have the capability of anchorage-independent growth, the cells were evaluated in soft-gel culture. The NEC and the sphere cells all formed significant number of colonies in soft agar gel, indicating that both cell types still retained tumorigenic potential. However, the number of colonies from the NEC (19.5 ± 5.9) was significantly lower than those from the spheres (46.5 ± 6.4) and the LNCaP cells (64.8 ± 7.7) (Figure 4B).

It is well known that carcinomas such as small cell lung cancer and small cell prostate cancer with neuroendocrine cell features metastasize early in the disease course. A Matrigel invasion chamber assay was used to examine the invasion capacity of the cells. In the presence of 10% regular FBS, the spheres had the lowest invasive activity with approximately 8.3 ± 1 invasive cells per membrane, as compared to the LNCaP cells, with 27.3 ±10.2 cells per membrane, and the NEC, with the highest activity at 37.6 ±12.3 cells per membrane (Figure 4C). The invasive activity of the NEC in the presence of charcoal-stripped FBS was approximately 32.6 ±1.2 cells per membrane, similar to that when regular FBS was used as chemoattractant. This indicates that the invasive activity of the NEC is independent of androgen. This property is consistent with the cell nature of these neuroendocrine cells which developed in the absence of androgen. The tumor cell type rather than the androgen level may be the determining factor for chemotaxic migration and invasion.

### Distinct epigenetic signatures of spheres

**Figure 5:**
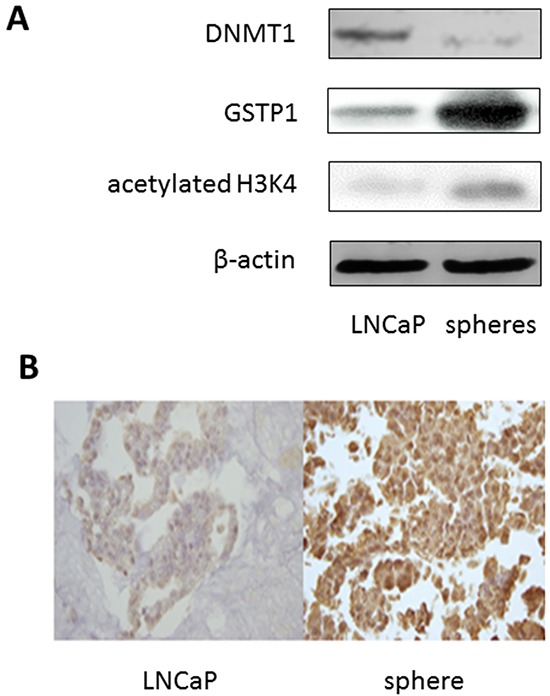
Epigenetic marks of LNCaP sphere cells **A.** Immunoblotting showing the expression levels of DNMT1 (DNA methytransferase 1), GSTP1 (π-class glutathione S-transferase), and acetylated histone H3 lysine 4 (H3K4) in LNCaP cells and sphere cells maintained in regular FBS medium. β-actin was used as a loading control. **B.** Immunohistochemical staining of GSTP1 on paraffin embedded preparations of LNCaP cells (left), and sphere cells (right) maintained in regular FBS medium.

Epigenetic marks in the spheres were compared with those in the LNCaP cells. LNCaP cells and the great majority of clinical prostate tumors are known to have the π-class glutathione S-transferase gene (GSTP1) silenced due to promoter hypermethylation [22, 23]. The expression levels of GSTP1 in the spheres after PEITC exposure were clearly increased (approximately 6.7 folds) in comparison to that of the untreated LNCaP cells (Figure 5A). Meanwhile, the expression level of DNA methyltransferase 1 (DNMT1), an enzyme responsible for DNA methylation, was significantly reduced in the spheres (approximately 5.7 folds), as compared to that of the parental LNCaP cells (Figure 5A). Figure 5A shows additionally that the level of acetylation of histone H3 lysine position 4 (H3K4) was increased (approximately 5 folds) in the spheres. Both the histone acetylation and DNMT1 changes were reported to be responsible for reactivating GSTP1 [15]. Activation of GSTP1 was further confirmed by *in situ* immunohistochemical staining of the sphere cells in paraffin blocks (Figure 5B). The sphere cells showed more intense staining. More sphere cells were positive for GSTP1 expression than LNCaP cells. The results indicated that the spheres had epigenomic signatures distinct from the parental LNCaP cells.

## DISCUSSION

This study established prostate cancer cell spheres through treatment with an epigenetic modulator PEITC. The sphere cells can self-renew in media with and without androgen, and both can be maintained as cell lines in long-term cultures. The phenotype of the LNCaP sphere cells is distinct from that of the parental LNCaP cells. The distinct characteristics of the sphere cells include the expression of CD44, higher expression levels of AR, Sp1, and PSA, as well as distinct epigenetic markers (Table 2). When androgen was depleted, the adherent spheres underwent differentiation to neuroendocrine cells. Like other types of tumor spheres, the LNCaP prostate cancer spheres have properties of cancer stem cells capable of self-renewal and differentiation [24–28]. Furthermore, the sphere formation property after PEITC treatment appears to be intrinsic to LNCaP cells, since sphere formation was reproducible in GFP-LNCaP cells, but no spheres from DU-145 cells and rare small spheres from PC-3 cells were observed under similar treatment. This may suggest that there may be unique epigenetic abnormalities in LNCaP cells.

**Table 2:** Comparison of the characteristics of LNCaP cells, spheres, and neuroendocrine cells

Markers and Properties	Characteristics		
	LNCaP	Spheres	Neuroendocrine cells
Pancytokeratin	+/−	−	+++
Neuro-specific enolase	+/−	-	+++
Serotonin	−	−	+
AR	++	+++	+
Sp1	+/−	+++	−
PSA	+	+++	−
Ki67	++	+++	+
CD44	<2%	46%	67%
Colony formation	+++	++	+
Invasive cells	++	+	+++
DNMT1	+++	++	+
GSTP1	+	++++	+++

Definitions: +/−: focally and weakly positive; −: negative; +: weakly positive; ++: moderately positive; >=+++: strongly positive.

Abbreviations: AR: androgen receptor; DNMT: DNA methyltransferase; GSTP: glutathione-S-transferase;

The current finding of the prostate cancer sphere cells is in line with earlier reports of androgen-independent prostate cancer stem cells [5, 6]. However, this is the first report of the dual capability of PCSC to self-renew with and without androgen. The results demonstrated that when the androgen quantity is decreased; the PCSC may reprogram to self-renew without androgen, and differentiate into progeny. The progeny could also reprogram and de-differentiate to PCSC when androgen is increased. Thus tumor cells may differentiate back and forth, capable of adapting to the microenvironment, growing according to the quantity of androgen.

**Figure 6:**
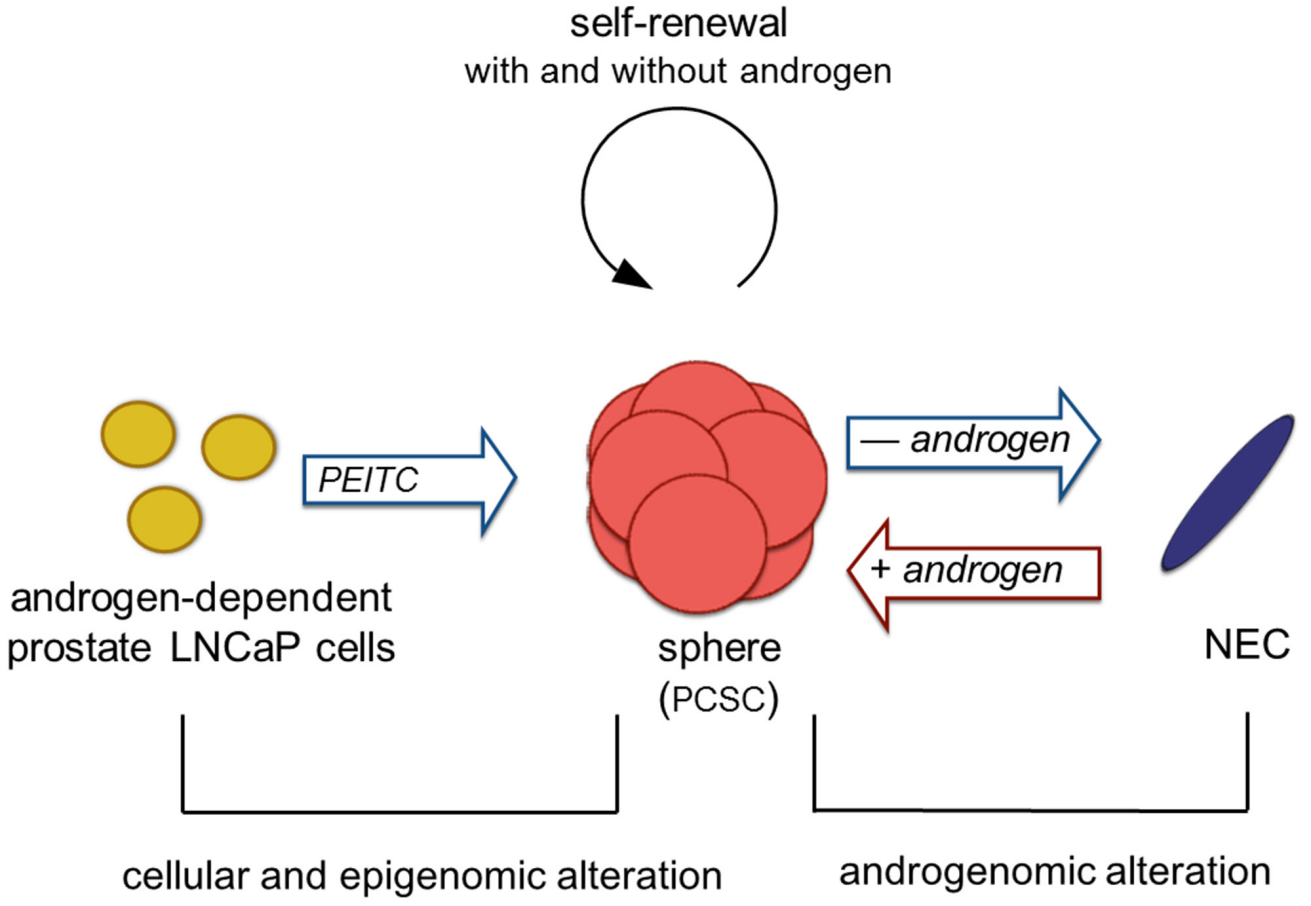
Hypothesis of a prostate cancer sphere model system Androgen-dependent prostate cancer cells form spheres, with characteristics of prostate cancer stem cells (PCSC), due to epigenomic alterations mediated by an epigenetic modulator PEITC. This step is irreversible. PCSC retain tumorigenic potential and have dual proliferation capabilities allowing self-renewal in the presence and absence of androgen. In the absence of androgen (such as complete androgen blockade therapy/castration), PCSC cells are attenuated in proliferation and initiated differentiation process to neuroendocrine cells (NEC). The androgenome in the NEC cells are reprogramed in the presence of androgen, thereby the NEC cells can de-differentiate to spheres. Since LNCaP cells are epithelial in origin, this step may represent a new phenomenon, epithelial-to-neuroendorine and neuroendocrine-to-epithelial transition.

The shuttle between PCSC and progeny differentiation may shed light on the drug resistance to androgen deprivation therapy. When the androgen-sensitive tumor cells are eliminated by androgen deprivation therapy, PCSC may be spared since they can survive in androgen insufficient conditions. Under low androgen concentration, PCSCs differentiate to progenies with low AR expression and survive in an androgen-depleted condition, thus becoming refractory to androgen deprivation therapy. The neuroendocrine cells, after differentiating from spheres in androgen deficient condition, were demonstrated to revert back to spheres upon addition of DHT, in a concentration-dependent manner. This indicates that the reversible process is androgen-dependent. However, the mechanism of de-differentiation from neuroendocrine cells to spheres remains unclear, as stem and progenitor cell differentiation is classically uni-directional. It has been shown that epithelial-to-mesenchymal transition (EMT) and vice versa can take place [29]. Whether the shuttling between spheres and neuroendocrine cells represents a new type of EMT (*i.e.*, epithelial to neuroendocrine transition) remains to be determined. We hypothesize that androgenomic alterations govern the shuttling between sphere PCSC and neuroendocrine cells (Figure 6). In the absence of androgen, such as complete androgen blockade therapy or castration, androgenomic shutdown in the PCSC may lead to deceleration in proliferation and self-renewal, and initiate the differentiation process to progeny such as neuroendocrine cells (NEC). In the presence of androgen, the androgenome in the NEC may reprogram, allowing de-differentiation to spheres and the regaining of their properties. Albeit rare, prostate cancer with neuroendocrine cell features is known to have an aggressive nature with a poor prognosis [9]. The NEC in this study had demonstrated tumorigenic and invasive activities, as well as cellular and molecular features resembling NEC in clinical tumors. The current culture system may serve as a model for investigating this population of cancer cells.

In comparison to the LNCaP cells, the sphere cells displayed distinct epigenetic marks at the two major epigenetic transcriptional controls, *i.e*., covalent modification of histones and DNA methylation. They include enhancement of acetylation at histone H3K4, and activation of hypermethylated GSTP1 gene. Exposure to PEITC was previously demonstrated to reactivate the GSTP1 gene and its detoxification function, by inhibiting histone deacetylases and promoter hypermethylation of the GSTP1 gene [15, 16]. PEITC might also regulate the molecules involved in cellular adherence, which resulted in detachment of PEITC-treated cells from flasks, leading to sphere formation in culture supernatant. The detailed mechanism of PEITC effect on these cells remains to be further investigated. They include whether PEITC causes specific changes in cellular organelle structure [30], and gene expression for PCSC development [31], and whether PEITC affects telomere stability, as AR is associated with telomeres [32]. An investigation of signaling pathways mediated by PEITC relating to sphere development is currently being pursued.

This study described a simple, unique and reproducible methodology for establishment and maintenance of prostate cancer spheres. The sphere cells have properties of PCSCs since they can self-renew and differentiate into neuroendocrine cells. Furthermore, the sphere cells can be manipulated to shuttle between spheres and neuroendocrine cells by adjusting androgen concentration. This has additionally described a method for establishment of a cell line that can shuttle between two distinct types of cells derived from the LNCaP cell line. This methodology might be applicable for establishment of spheres from other cell lines. More and more biomarkers are being discovered and validated for clinical diagnosis and therapeutic development [33–36]. Targeted therapy with small molecule inhibitors are translated rapidly to clinical applications [37–43]. The PCSC sphere cell line established herein can thus be used to screen for novel agents for prostate cancer therapy.

## MATERIALS AND METHODS

### Cell lines and reagents

Human prostate cancer LNCaP cell line at passage 21 and 31 were purchased from ATCC and characterized for fewer than 6 months before experimentation. An LNCaP cell line labeled with green fluorescent protein (GFP-LNCaP) was purchased from AntiCancer, Inc. (San Diego, California). LNCaP cells were maintained in RPMI-1640 medium supplemented with either 10% fetal bovine serum (regular FBS), or with 10% steroid hormone-depleted charcoal dextran-stripped FBS (Hyclone) (CSF), and antibiotics. 5-αdihydrotestosterone (DHT) was purchased from Sigma Chemical Company.

### Immunohistochemistry

Cultured cells were fixed in 10% neutral buffered formalin, and the centrifuged cells underwent routine paraffin-embedding as a cell block by the plasma thrombin method. Three to four micrometer sections of the paraffin-embedded cell blocks were prepared for histology study and for immunohistochemistry. Monoclonal antibodies for the following proteins were used: pan cytokeratin (Ventana; AE1/AE3/PCK26); serotonin (Dako; 5HT-H209, 1:5 dilutions); neuro-specific enolase (Ventana; MRQ-55); Ki67 (Ventana; 30-9); androgen receptor (Ventana; SP107); π-class glutathione S-transferase (Santa Cruz biotechnology; A1513). Automated immunohistochemical system Ventana Bench Mark Ultra was used. Ventana ultra detection kit was used for staining and visualization. Positive controls included known pan cytokeratin, neuro-specific enolase, serotonin, Ki67, AR, and π-class glutathione S-transferase. Negative controls had primary antibody replaced by buffer and processed with the specimen slides. Immunohistochemistry was performed at the Department of Pathology, Westchester Medical Center, and immuostaining evaluated by an intensity scoring procedure. Cells were graded for the staining intensity as negative (-), focal and weak (±), weak to moderate (+), or strong (+++). Evaluations of specimen staining were performed by an independent pathologist without knowing the nature of the experiments.

### LNCaP cell sphere development

LNCaP cells seeded at 1.5 × 10^5^/ml of RPMI-1640 medium with 10% regular FBS medium were exposed to 4 μM phenethyl isothiocyanate (PEITC). PEITC was supplemented every 2-3 days. PEITC was purchased from LKT Labs with 98% purity, which was prepared in phosphate buffered saline and DMSO [15, 16]. Cultures supplemented with vehicle control were used in every experiment. The non-adherent cells in the supernatants, after exposure to PEITC for 4-10 days, were collected in regular culture flasks, which were continuously exposed to 3 μM PEITC for 8-12 days. The non-adherent spheres were collected by centrifugation, and cultured in flasks with ultralow attachment surface (Fisher Scientific). They were maintained in RPMI-1640 medium with 10% FBS without PEITC for further studies. Adherent sphere culture was obtained by culturing the floating spheres in regular culture flasks. Individual spheres were isolated with limiting dilution in 96 well plates [44]. Dissociation of cells from spheres was aided with a non-enzymatic cell dissociation solution (Sigma).The cells were further dispersed by repetitive pipetting through a glass Pasteur pipette, in combination with trypsin digestion and gently passing through 17 G, and 25 G needles.

### Cell cycle analyses and immunoblotting

Cell cycle phase progression was measured by a BD FACS Calibur flow cytometer with the method described previously [45, 46]. The cells were fixed with 80% ethanol at 4°C, and incubated on ice before the DNA was stained with propidium iodide (50 μg/ml). The expression levels of cellular proteins were determined by quantitative Western blotting as previously described [47, 48]. Images were recorded using chemilmager 5500 (Alpha Innotech). Antibodies were used to detect the following: C-terminal region of the androgen receptor (AR) (Santa Cruz Biotechnology;I299), Sp1 (Santa Cruz Biotechnology; E1304), prostate specific antigen (PSA) (Santa Cruz Biotechnology; C0414), DNA methytransferase 1 (DNMT1) (Santa Cruz Biotechnology; C0306), π-class glutathione S-transferase (GSTP1) (Santa Cruz Biotechnology; A1513), and β-actin (Sigma; 082M4781). The relative abundance of each protein obtained from the blot was calculated relative to the quantity of β-actin. Cells with surface CD44 were detected by a flow cytometric method with 10,000 live cell events analyzed per sample [49]. Cells were immunostained with a fluorescent conjugated monoclonal antibody against CD44 (BD Biosciences; clone 24345), and fixed with 1% paraformaldehyde. The proportion of the positively stained cells was determined against a background of cells stained with an isotype control.

### Clonogenesis and cell invasion assay

Clonogenesis of cancer cells was assayed by a soft-gel semi-solid culture as previously described [49]. Essentially, 400-800 single cells were seeded in 1.5 ml soft-agar gel with RPMI-1640 medium with either 10% regular FBS or charcoal-stripped FBS per 35 × 10-mm culture dish. At least five duplicate dishes were used for each condition. Individual colonies with at least 30 cells were enumerated. Cell invasion assay was performed using a BD BioCoat Matrigel Invasion chamber (BD Biosciences) according to the procedure described. The upper chambers were seeded with 5 × 10^4^ of LNCaP cells, dissociated cells from the spheres, or neuroendocrine cells which were scraped from the cultures. Chemoattractants were loaded in the lower chamber. These included 10% FBS, or 10% charcoal–stripped FBS as indicated. Triplicate samples were assayed. Cells migrated into the lower chamber were fixed, stained with 1% toluidine blue and counted under light microscope.

### Statistical analysis

All data were presented as mean ± SD from multiple independent experiments. The mean values of two groups of data were compared by the two-tailed Student's t-test, with *P*<0.05 considered to be statistically significant.

## SUPPLEMENTARY TABLE


