# Supplementary material for: Establishment of prostate cancer spheres from a prostate cancer cell line after phenethyl isothiocyanate treatment and discovery of androgen-dependent reversible differentiation between sphere and neuroendocrine cells
Source: Oncotarget. 2016 Mar 28;7(18):26567–79. doi: 10.18632/oncotarget.8440 (PMC5041999; doi:10.18632/oncotarget.8440)
Supplement: Supplementary file 1 [file oncotarget-07-26567-s001.pdf]

SUPPLEMENTARY TABLE

Supplementary Table S1: Sphere formation assays on prostate cancer cell lines

cell lines	spheres	PEITC (μM)	exposure (days)
LNCaP	++++	4	4-7
GFP-LNCaP	++++	4	4-7
PC-3	+/−	2	8-12
DU-145	−	2-4	4-10

Sphere formation assay was performed with several prostate cancer cell lines (table S1). We explored a variety of assay conditions, including the length of exposures to PEITC and PEITC concentrations. Details on LNCaP and GFP-LNCaP cell lines were in the official text. DU-145 failed to show any sphere formation. PC-3 had extremely low efficiency of sphere formation (number of spheres from number of cells placed in culture), with more irregular-shaped spheres when the spheres did show up. GFP-LNCaP cell line was obtained from an independent commercial source (see Material and Methods). The cell line was established by inserting GFP into the LNCaP cells, and therefore should be considered as a separate sub-cell line of LNCaP. This GFP-LNCaP sub-cell line had the same sphere formation efficiency like the parental cell line. Using this cell line, we also proved with GFP staining that the neuroendocrine differentiation was indeed from the parental LNCaP cells.
